# Endoscopic closure of a persistent tracheoesophageal fistula using a cardiac occluder device

**DOI:** 10.1055/a-2877-1875

**Published:** 2026-05-29

**Authors:** Ahmed Alwali, Paul Stoll, Clemens Schafmayer, Imad Kamaleddine

**Affiliations:** 1 Department of General, Visceral, Thoracic, Vascular and Transplant Surgery 39071 Rostock University Medical CenterRostockMVGermany; 2 Department of Pneumology and Critical Care Medicine 39071 Rostock University Medical CenterRostockGermany

**Keywords:** Other focus (of reviewers), GI surgery, Laparoscopy, Endoscopic ultrasonography, Esophageal cancer

A tracheoesophageal fistula (TEF) is a rare but severe late complication after esophagectomy, associated with recurrent aspiration and significant morbidity. Endoscopic treatment options include stenting, clipping, suturing, and endoscopic vacuum therapy; however, durable closure remains challenging in refractory cases.

We report a 68-year-old patient who developed a persistent TEF 6 years after neoadjuvant radiochemotherapy and transthoracic esophagectomy for squamous cell carcinoma. The patient presented with progressive dysphagia, recurrent aspiration, and repeated episodes of bronchitis, leading to a significant impairment in quality of life.

Computed tomography and endoscopic evaluation confirmed a chronic fistula. Gastroscopy demonstrated an approximately 8 mm defect at 22 cm from the upper incisors, while bronchoscopy identified the corresponding opening approximately 1 cm proximal to the carina ( Fig. 1 ).

**Fig. 1:**
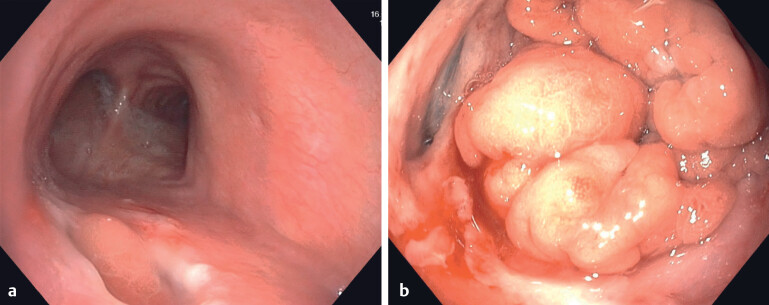
( **a** ) A bronchoscopic view demonstrating a fistula proximal to the carina. ( **b** ) A gastroscopic view showing the fistula at the esophagogastric anastomosis.

A combined therapeutic approach was initially attempted, including tracheal stent placement, endoscopic suturing using a needle holder and a helical tacking system, and prior intraluminal endoscopic vacuum therapy. Despite technically successful interventions and partial granulation, contrast imaging revealed persistent leakage, and repeat endoscopy showed dehiscence of the suture line ( Fig. 2 ).

**Fig. 2:**
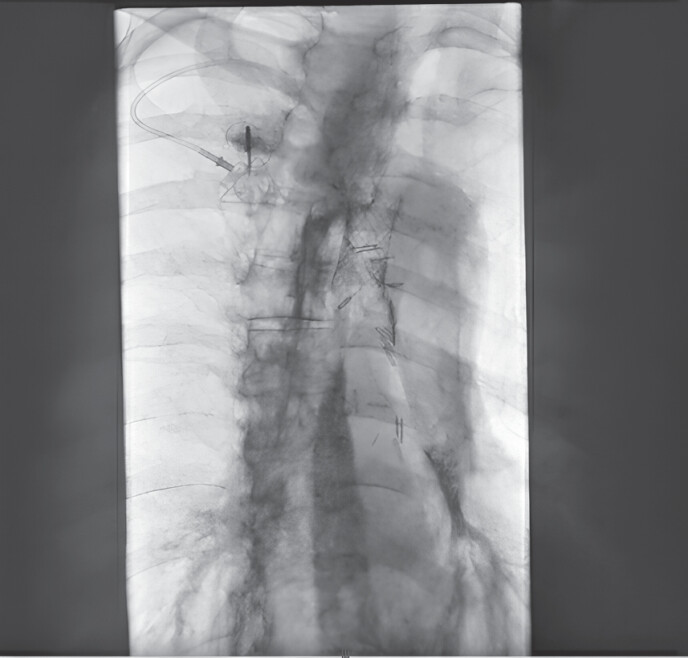
The tracheal stent in situ; contrast imaging demonstrated persistent leakage into the tracheobronchial system, indicating ongoing fistula patency.

Given treatment failure and the high operative risk in previously irradiated tissue, a multidisciplinary decision was made to proceed with off-label closure using a cardiac septal occluder ( Video 1) . Under general anesthesia, the tracheal stent was removed, resulting in the enlargement of the fistula to approximately 11 mm. A guidewire was advanced through the defect under combined gastroscopic and bronchoscopic guidance. The occluder was then deployed transesophageally, with the proximal disc (25 mm) released in the trachea and the distal disc (30 mm) in the esophagus, achieving complete sealing ( Fig. 3 ). Notably, despite the enlargement of the defect after stent removal, secure closure was achieved using an oversized device **,** allowing stable anchoring in fibrotic tissue.

**Video 1** Endoscopic closure of a tracheoesophageal fistula using a cardiac septal occluder.


**Fig. 3:**
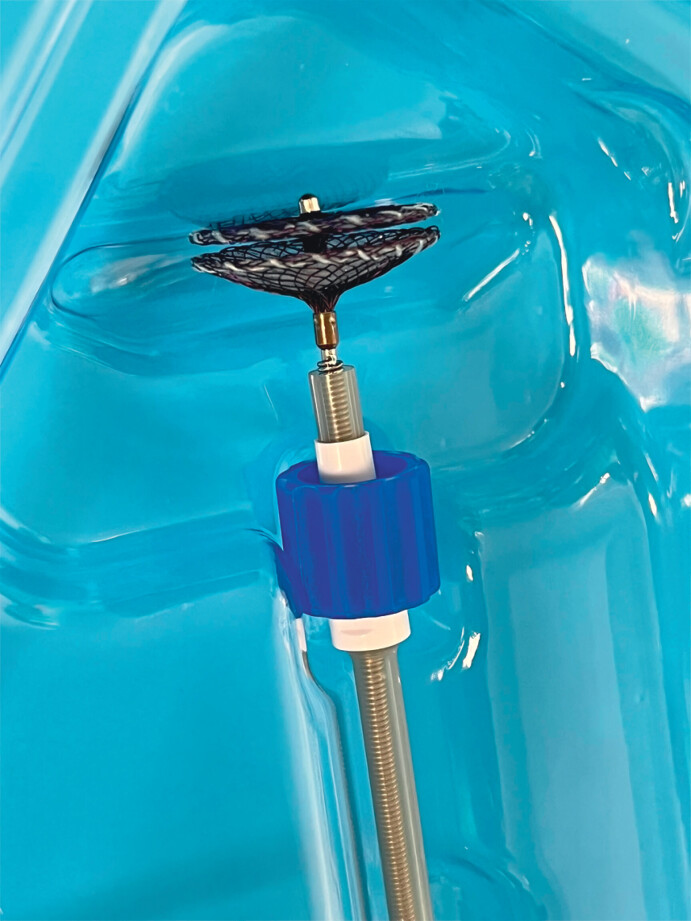
The PFO occluder (25/30 mm). We recommend choosing an occluder that provides at least 8–10 mm of circumferential overlap beyond the fistula margins to ensure stable positioning and effective sealing. PFO, patent foramen ovale.

Postprocedural fluoroscopy confirmed closure without leakage. Clinically, aspiration resolved. At the 4-month follow-up, imaging showed stable device position without recurrence ( Fig. 4 ).

**Fig. 4:**
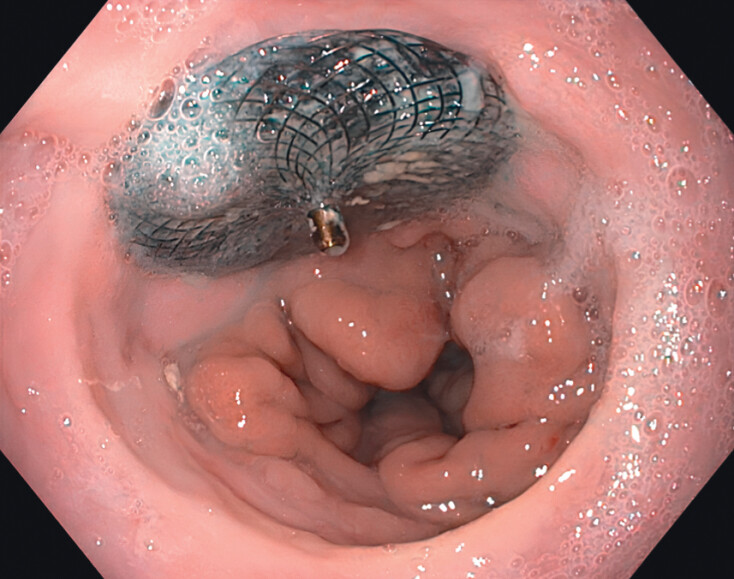
A gastroscopic view showing the PFO occluder in situ 4 months after intervention. PFO, patent foramen ovale.

Endoscopic closure of the TEF using a cardiac occluder is a feasible minimally invasive salvage option after failure of conventional therapies, particularly in high-risk surgical patients.

Endoscopy_UCTN_Code_TTT_1AO_2AI

## Informed Consent

The patient provided a written informed consent for the anonymous publication of their medical data, videos and images.

